# Toward clinical translation of TMS-EEG: an integrative review of multidimensional neurophysiological measures

**DOI:** 10.3389/fnhum.2026.1804482

**Published:** 2026-06-19

**Authors:** Wei Wang, Yuyao He, Haoyang Xing, Hongmei Zhang

**Affiliations:** 1 The Second Veterans Hospital of Sichuan, Chengdu, China; 2 Medical Imaging Laboratory, College of Physics, Sichuan University, Chengdu, China; 3 Huaxi MR Research Center (HMRRC), Department of Radiology, West China Hospital of Sichuan University, Chengdu, China

**Keywords:** clinical translation, global mean field power, time-frequency analysis, TMS-EEG, TMS-evoked potentials

## Abstract

Transcranial magnetic stimulation combined with electroencephalography (TMS-EEG) has emerged as a promising noninvasive approach for probing cortical reactivity and network dynamics with millisecond temporal resolution, with growing relevance for mechanistic and clinical neuroscience. However, despite rapid expansion of the field, TMS-EEG findings remain difficult to interpret because of substantial inter-individual variability and fragmentation across distinct analytical domains, including time-domain, spatially integrated, and time-frequency measures. These issues limit cross-study comparability and hinder clinical translation. In this review, we summarize the conceptual basis of TEPs, the physiological interpretation of canonical components (e.g., N15, P30), and commonly adopted analytical considerations. We further synthesize spatially integrated measures, including global and local mean field power (GMFP and LMFP), which quantify distributed cortical responses across multiple electrodes. Time-frequency indices, such as time-related spectral perturbation (TRSP) and inter-trial phase coherence (ITPC), are also reviewed to characterize TMS-induced oscillatory modulations. Drawing on the existing TMS-EEG literature in humans, we outline representative applications of these multidimensional metrics in neuropsychiatric and neurological conditions, including Alzheimer’s disease, depression, epilepsy, and stroke. Finally, we discuss key methodological and translational challenges, such as stimulation-related artifacts, inter-individual variability, state-dependency of baseline oscillatory activity, and limited standardization, and highlight how these issues complicate the interpretation of TMS-EEG findings across studies and individuals. Together, this review aims to provide a structured reference framework for integrating and interpreting multidimensional TMS-EEG measures, thereby supporting more consistent understanding of the literature and informing future efforts toward harmonization and clinical translation.

## 1 Introduction

Transcranial magnetic stimulation combined with electroencephalography (TMS-EEG) is increasingly recognized as a promising tool for translational neuroscience because it enables direct, noninvasive assessment of cortical reactivity and large-scale network dynamics with millisecond temporal resolution. At the same time, the growing TMS-EEG literature remains methodologically and conceptually fragmented: time-domain responses, spatially integrated indices, and time-frequency measures are often discussed in parallel rather than within a unified interpretive framework. Moreover, TMS-EEG outcomes can vary substantially both within and between individuals, reflecting differences in cortical anatomy, ongoing brain state, and other biological factors. In such a context, fragmented analytical interpretation further complicates cross-study comparison and makes it more difficult to distinguish meaningful individual differences from inconsistencies related to divergent methodological practices. Against this background, there is a clear need for an integrative review that brings together these multidimensional neurophysiological measures and provides a more coherent framework for interpreting their physiological meaning, methodological limitations, and potential clinical utility.

TMS-EEG integrates non-invasive brain stimulation with high-temporal-resolution electrophysiological recording. By delivering brief magnetic pulses to targeted cortical regions while simultaneously recording phase-locked EEG responses, TMS-EEG captures TMS-evoked potentials (TEPs) and enables direct characterization of local cortical excitability and the propagation dynamics of stimulation effects across large-scale brain networks on a millisecond timescale (Ilmoniemi and Kičić, 2010; Tremblay et al., 2019). Compared with resting-state EEG or motor-evoked potentials (MEPs) elicited by TMS, TEPs do not rely on peripheral output pathways and therefore provide a more direct measure of causal cortical responses.

From a neurophysiological perspective, TEPs arise from the combined effects of excitatory and inhibitory postsynaptic potentials following stimulation. Distinct TEP components at different latencies are thought to reflect the activation of local cortical circuits as well as trans-synaptic and interregional connectivity processes (Kirschstein and Köhling, 2009; Farzan et al., 2016). Accordingly, TEPs have been widely regarded as sensitive markers of cortical excitation-inhibition (E/I) balance and network reactivity, and have been increasingly applied in mechanistic studies and treatment evaluations across a range of neuropsychiatric disorders.

However, the reliable extraction and interpretation of TEPs critically depend on data quality and analytical methodology. Non-neural artifacts, including coil click–related auditory responses, scalp and temporalis muscle contractions, electrode saturation, and interactions between the stimulation pulse and EEG amplifiers, can substantially contaminate early and mid-latency TEP components. Moreover, the choice of artifact-removal strategies and preprocessing parameters may introduce systematic biases across studies (Beck, Heyl et al., 2024). Previous work has demonstrated that differences in preprocessing pipelines alone can result in systematic variations of approximately 30–50% in estimated TEP amplitudes, posing significant challenges for cross-study comparison and reproducibility. These issues become even more important in a field where considerable inter-individual variability is already expected, because analytical inconsistency may obscure rather than clarify variability that is truly attributable to individuals.

In this context, reliance on single electrodes or isolated TEP components is often insufficient to comprehensively characterize TMS-induced cortical responses. To improve robustness and comparability, spatially integrated metrics that aggregate multichannel signals, such as global mean field power (GMFP) and local mean field power (LMFP), have been increasingly adopted to complement conventional TEP analyses, thereby reducing the influence of random noise and electrode-selection bias. In parallel, frequency-domain and time-frequency approaches have expanded the analytical scope of TMS-EEG, including TMS-related spectral perturbation (TRSP) and inter-trial phase coherence (ITPC), which allow more comprehensive characterization of stimulation-induced oscillatory dynamics and their potential modulation of E/I mechanisms.

In light of these considerations, this review focuses on human TMS-EEG studies, excluding animal experiments and purely computational simulations. The literature discussed here was identified primarily through PubMed and Web of Science up to October 2025, using key terms such as “TMS-EEG,” “TMS-evoked potentials,” “TEP,” and related combinations, with additional relevant studies identified through reference screening. Within this scope, the review emphasizes work on TEPs, spatially integrated measures such as GMFP and LMFP, time-frequency indices, and their applications in Alzheimer’s disease, depression, epilepsy, and stroke, and discusses current methodological and translational challenges, particularly with regard to artifacts, inter-individual variability, limited standardization, multimodal integration, and the need for larger controlled studies. Overall, we aim to provide an integrative framework to support both mechanistic research and the clinical translation of TMS-EEG.

## 2 Time-domain analysis

### 2.1 TMS-evoked potentials (TEPs)

#### 2.1.1 Definition

TEPs constitute the most fundamental and intuitive time-domain measure in TMS-EEG research. They refer to a sequence of phase-locked voltage deflections recorded in the EEG following single-pulse or paired-pulse TMS, reflecting the direct cortical response to external magnetic stimulation (Ilmoniemi and Kičić, 2010; Tremblay et al., 2019). Unlike conventional event-related potentials (ERPs), which are passively elicited by sensory stimuli, TEPs arise from the direct activation of targeted cortical regions and therefore provide a causal measure of local cortical reactivity and its propagation to remote brain areas.

From a methodological perspective, however, the extraction and quantification of TEPs are highly sensitive to experimental and analytical choices. Parameters such as stimulation intensity, coil orientation, ongoing brain state, and the selection of artifact-removal strategies, particularly the use of independent component analysis (ICA), can exert systematic effects on TEP morphology and amplitude. As a result, substantial variability in reported TEP amplitudes has been observed across studies, even when similar stimulation paradigms are employed. This methodological sensitivity limits;direct comparability between studies and necessitates caution when interpreting single TEP components in isolation.

Consequently, reliance on individual electrodes or isolated TEP peaks may be insufficient for robust cross-study integration. This limitation has motivated the progressive adoption of multi-metric and multilevel analytical frameworks that complement conventional TEP analysis, aiming to enhance the stability, interpretability, and reproducibility of TMS-EEG findings.

#### 2.1.2 Characteristics and canonical components

**Figure 1:**
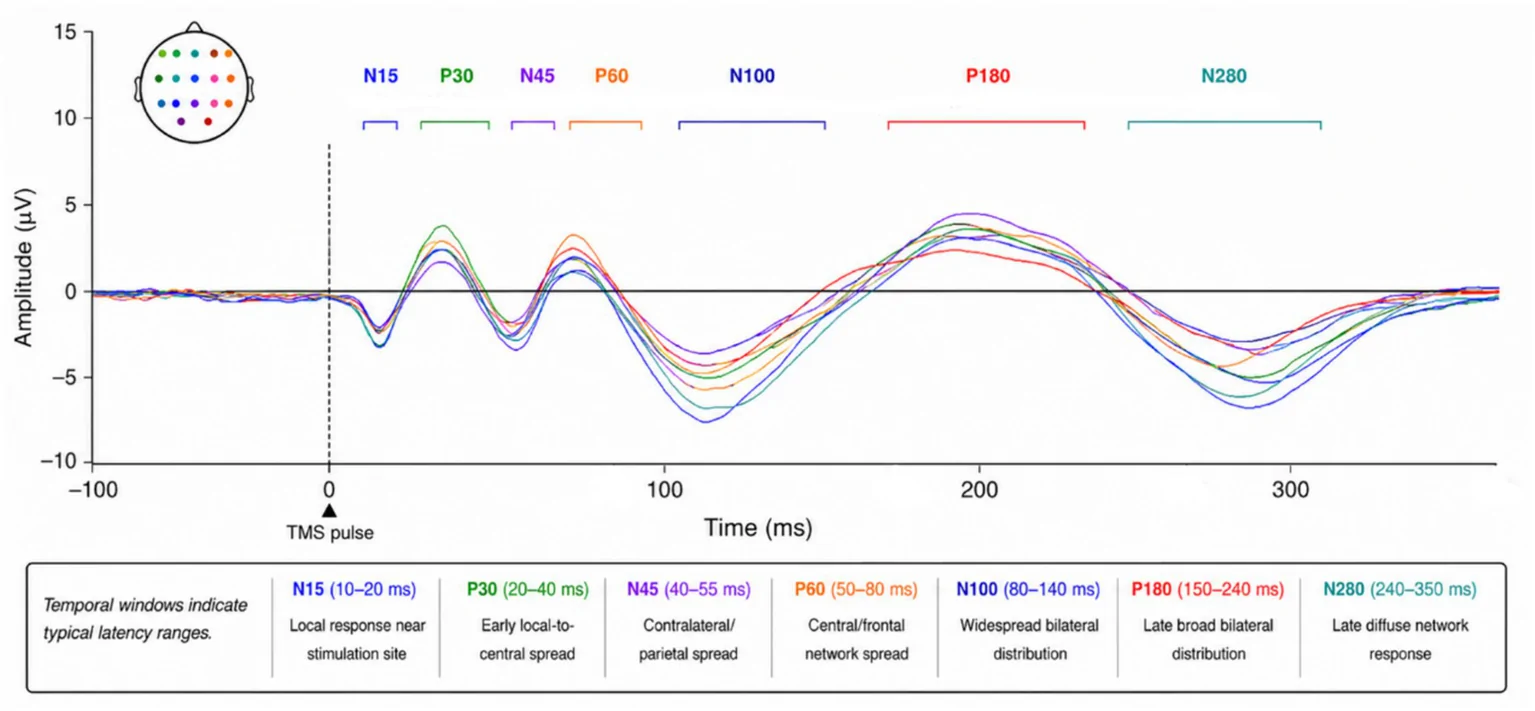
Schematic illustration of canonical TMS-evoked potential components. The figure illustrates the typical temporal sequence of TEP components following single-pulse TMS, including N15, P30, N45, P60, N100, P180, and N280. It is provided as a conceptual illustration to support interpretation of the canonical time-domain structure of TEPs. Original schematic illustration created by the authors for this review.

The waveform characteristics of TEPs are jointly influenced by stimulation site, stimulation intensity, and inter-individual neurophysiological variability. When TMS is applied to M1, a sequence of phase-locked cortical responses lasting approximately 300 ms can be observed not only in the vicinity of the stimulation site but also in distal, functionally connected regions. Across different experimental paradigms, a set of representative TEP components has been consistently identified, temporally organized in a sequential manner and thought to reflect successive stages of cortical excitation, inhibitory regulation, and higher-order network-level information processing. To provide a clearer overview of this temporal organization, Figure 1 schematically illustrates the typical sequence of canonical TEP components, including N15, P30, N45, P60, N100, and P180. This figure is intended to help readers follow the subsequent discussion of individual components and their putative neurophysiological interpretations.

N15 is the earliest negative TEP component, typically observed within the 10–20 ms post-stimulation window and primarily localized near the stimulation site (Tremblay et al., 2019; Rogasch and Fitzgerald, 2013). It is generally interpreted as reflecting an early cortical response directly induced by TMS, potentially associated with the initial recruitment of local neuronal populations (Ilmoniemi and Kičić, 2010; Tremblay et al., 2019). However, TMS-EEG signals within the first 30 ms are especially susceptible to stimulation-related artifacts, and short-latency components in this time range should therefore be interpreted cautiously when artifact control is incomplete (Rogasch et al., 2013; Rogasch and Fitzgerald, 2013; Hernandez-Pavon et al., 2023). Direct pharmacological evidence supporting a specific receptor-level interpretation of N15 remains limited. Some studies of short-latency TEP modulation, including work using theta-burst paradigms, suggest that early components may relate to baseline cortical excitability, although this interpretation remains tentative (Veniero et al., 2013).

P30 is one of the most reproducible early positive TEP components, emerging approximately 20–40 ms after stimulation and often spreading from the stimulation site toward central regions. It is generally interpreted as reflecting early cortical excitability and rapid recruitment of local circuits, although its physiological specificity remains less firmly established than that of later components such as N45 or N100 (Tremblay et al., 2019; Beck, Heyl et al., 2024). Pharmaco-TMS-EEG studies suggest that early TEPs below 50 ms are sensitive to GABA_A-ergic modulation, but the clearest receptor-level evidence within this latency range has been reported for N45 rather than for P30 specifically (Premoli et al., 2014; Tremblay et al., 2019). In Alzheimer’s disease and mild cognitive impairment, P30 amplitude has been associated with cognitive decline and has shown discriminatory value between healthy individuals and cognitively impaired groups in small-sample studies, supporting its potential relevance as an early marker of altered cortical reactivity (Julkunen et al., 2011).

N45 typically appears within the 40–55 ms post-stimulation interval and often propagates toward contralateral and parietal regions (Tremblay et al., 2019; Rogasch and Fitzgerald, 2013). Among canonical TEPs, N45 has some of the clearest links to GABA_A-mediated inhibitory neurotransmission: benzodiazepine-type positive modulators such as alprazolam and diazepam increase N45 amplitude, and the selective α5-GABA_A antagonist S44819 has also been shown to alter this component (Premoli et al., 2014; Darmani et al., 2016). Paired-pulse TMS-EEG studies further demonstrated that long-interval intracortical inhibition (LICI) suppresses N45 together with several other canonical peaks, supporting the relevance of N45 to inhibitory cortical physiology (Premoli et al., 2014; Tremblay et al., 2019). However, the interpretation of N45 as a purely GABA_A-specific marker should be treated with caution. Recent pharmaco-TMS-EEG evidence indicates that NMDA receptor antagonism can also increase N45 amplitude, suggesting that this component may reflect a broader balance between GABA_A-mediated inhibition and glutamatergic excitation rather than a single receptor-specific process.

P60 reflects a further integration stage of excitatory and inhibitory processes and commonly extends to central and contralateral frontal regions. In paired-pulse paradigms such as short-interval intracortical inhibition (SICI) and intracortical facilitation (ICF), P60 amplitude shows bidirectional modulation (Tremblay et al., 2019; Rogasch and Fitzgerald, 2013). Low-frequency (1 Hz) rTMS has been reported to alter P60 amplitude, although its physiological interpretation remains less specific than that of N45 or N100 (Cruciani et al., 2023; Tremblay et al., 2019). Clinically, P60 has been used to assess cortical reactivity and connectivity in conditions such as epilepsy and mTBI, although its interpretation requires attention to paradigm-specific factors and potential sensory contamination (Cruciani et al., 2023; Bonato et al., 2006). Although pharmacological modulation involving both GABA_A- and GABA_B-related mechanisms has been discussed, P60 is also susceptible to peripheral sensory contamination, and its neural interpretation should therefore be contextualized within specific experimental conditions (Tremblay et al., 2019; Bonato et al., 2006; Cruciani et al., 2023).

N100 is among the most extensively studied and reliable TEP components, typically exhibiting a bilateral scalp distribution. Pharmacological and paired-pulse TMS-EEG studies have commonly related this component to slower inhibitory processes, particularly GABA_B-mediated mechanisms, as baclofen has been shown to enhance N100 amplitude and LICI paradigms strongly modulate late TEP responses (Premoli et al., 2014; Premoli et al., 2017; Tremblay et al., 2019). At the same time, the physiological interpretation of N100 requires caution because this component emerges within a latency range that overlaps with auditory and somatosensory evoked responses. Its amplitude may therefore be influenced not only by inhibitory cortical processing, but also by stimulation site, vigilance and attentional state, sensory co-activation, and preprocessing procedures. In line with this view, optimized sham-controlled TMS-EEG work using a GABAergic challenge reported that diazepam-related modulation of N100 was observed in both active and sham conditions, suggesting that sensory-evoked activity may contribute substantially to this late component (Gordon et al., 2023). Thus, N100 should be regarded as a robust but physiologically composite marker of late cortical and sensory-network processing, rather than as a specific readout of GABA_B receptor activity.

P180 occurs within a broad 150–240 ms post-stimulation window and is generally interpreted more cautiously than earlier canonical peaks (Rogasch and Fitzgerald, 2013; Tremblay et al., 2019). Because TMS coil discharge generates a loud click that activates auditory pathways through both air and bone conduction, late positive activity in this latency range is likely to overlap with the auditory N100–P180 complex and should not be interpreted as a purely local cortical response (Nikouline et al., 1999; Lioumis et al., 2009; Tremblay et al., 2019). P180 may therefore reflect a mixture of later sensory processing and network-level activity, and careful control of auditory co-activation is required when interpreting this component in TMS-EEG studies (Bonato et al., 2006).

N280 is a later negative component usually observed approximately 240–350 ms after stimulation and is discussed less frequently than earlier canonical peaks (Rogasch and Fitzgerald, 2013; Tremblay et al., 2019). Current evidence supports a cautious interpretation of N280 as a late large-scale or multimodal response rather than a component with well-established receptor-level specificity. For this reason, N280 is better treated as a descriptive late TEP component whose physiological meaning remains less clearly defined than that of N45 or N100.

Overall, pharmacological TMS-EEG studies provide a valuable basis for linking canonical TEP components to inhibitory and excitatory neurotransmission, but the physiological meaning of these components remains context-dependent. A drug-induced change in a specific peak should be interpreted as evidence that the underlying circuit is sensitive to that pharmacological manipulation, rather than as proof that the peak uniquely indexes one receptor system. This point is illustrated by N45, which has been widely related to GABA_A-mediated inhibition, while also being modulated by NMDA receptor antagonism, indicating that it may reflect the dynamic balance between inhibitory and excitatory synaptic activity (Premoli et al., 2014; Belardinelli et al., 2021). Likewise, N100 is often discussed in relation to GABA_B-mediated inhibition, but its late latency makes it particularly susceptible to auditory and somatosensory contributions; optimized sham-controlled evidence further suggests that GABAergic modulation of N100 may partly involve sensory-evoked activity (Premoli et al., 2014; Gordon et al., 2023). For this reason, TEP components should be interpreted within the broader experimental and clinical context, including stimulation site, participant state, sensory control, and preprocessing strategy, rather than being treated as isolated receptor-level biomarkers.

The limitations summarized above further indicate that TEP components should be interpreted within a broader experimental and biological context, rather than solely through pharmacological associations. Their amplitude and latency are influenced by multiple factors, including stimulation intensity, coil type, stimulation site, and inter-individual differences in cortical anatomy and functional organization. For example, compared with subthreshold stimulation, suprathreshold TMS typically induces more pronounced middle- and late-latency responses, particularly reflected by increased N45 and N100 amplitudes, whereas subthreshold stimulation often elicits relatively small early responses (Beck, Heyl et al., 2024). In addition, differences in coil geometry, pulse waveform, current direction, and target region may alter both local cortical activation and subsequent propagation across connected networks (Tremblay et al., 2019; Beck, Heyl et al., 2024). TEPs may also be confounded by non-neural signals, including stimulation-induced somatosensory feedback, scalp muscle activity, and auditory responses evoked by the coil click. These confounds necessitate careful experimental design, appropriate sensory control, and rigorous preprocessing procedures to disentangle neural from non-neural contributions.

#### 2.1.3 Data analysis methods

Reliable extraction and quantitative analysis of TEPs depend on careful EEG preprocessing and analysis choices, with the primary objective of improving signal-to-noise ratio while enhancing the stability and interpretability of results. In general, TMS-EEG data analysis involves several key steps.

During data acquisition, high-density EEG systems are commonly employed to record TMS-induced cortical responses, thereby enhancing spatial sampling and increasing sensitivity to the propagation of stimulation effects to remote cortical regions. To reduce the influence of random noise on single-trial responses, multiple trials are typically averaged, with the number of trials generally ranging from 50 to 200, depending on stimulation site, stimulation intensity, and artifact burden (Tremblay et al., 2019).

Because TMS-EEG signals are highly susceptible to contamination by non-neural components, artifact suppression represents a critical step in the analysis pipeline. Common artifacts include transient saturation artifacts induced by the stimulation pulse, electromyographic artifacts from scalp and temporalis muscle contractions, ocular artifacts, and auditory responses elicited by coil click sounds. Whenever possible, these sources of contamination should also be mitigated during data collection through careful participant setup, reduction of muscle activation, auditory masking or noise control, and stable recording conditions. Current studies typically combine multiple approaches for artifact correction and suppression, such as independent component analysis (ICA), signal space projection (SSP), and noise masking, in order to maximally remove artifacts while preserving genuine neural signals (Cruciani et al., 2023).

Regarding stimulation parameter settings, stimulation intensity is usually individualized based on the RMT to ensure reliable cortical responses while maintaining safety. Consistent control of coil orientation and position is also important at the acquisition stage, and an increasing number of studies employ MRI-guided neuronavigation systems to improve targeting accuracy and reduce variability related to stimulation delivery (Beck, Heyl et al., 2024).

It should be noted that substantial heterogeneity persists across current TMS-EEG studies with respect to preprocessing strategies, artifact-correction methods, and parameter selection. Different analysis pipelines can exert systematic effects on TEP amplitude estimates and temporal characteristics, thereby limiting reproducibility and cross-study comparability. Consequently, improving transparency and consistency across laboratories remains an important challenge in the TMS-EEG field.

#### 2.1.4 Clinical and research applications

As a non-invasive tool for assessing cortical function and network reactivity, TEPs have been widely applied in both basic neuroscience research and clinical studies of neuropsychiatric disorders, primarily in the characterization of disease mechanisms and the evaluation of neuromodulatory effects.

In Alzheimer’s disease (AD) research, TMS-EEG has been used to assess cortical reactivity of the precuneus, revealing local cortical hyperexcitability in AD patients, manifested as significantly increased TEP amplitudes. In these studies, TEPs were quantified as indices of cortical excitability and further correlated with cerebrospinal fluid biomarkers, supporting their potential utility as electrophysiological biomarkers of AD-related cortical hyperexcitability (Casula et al., 2023). In depression research, changes in the amplitude of the N100 component following DLPFC stimulation have been shown to predict rTMS treatment response, suggesting that TEPs may index inhibitory cortical function and sensitivity to neuromodulatory interventions in patients with major depressive disorder (Sheen et al., 2024). Evidence from motor-learning paradigms further extends this view, showing that M1-evoked N100 amplitude may be related to subsequent motor adaptation and learning-related plasticity (Taga et al., 2021).

These functional readouts should also be considered in relation to the structural substrate of the stimulated cortex. TMS-evoked responses are generated within cortical tissue whose morphology, laminar organization, and developmental history may differ across individuals and clinical populations. Recent large-scale structural MRI studies in youth have shown both shared and disorder-specific cortical thickness alterations across internalizing, externalizing, and thought disorders, suggesting that psychiatric vulnerability is accompanied by measurable variation in cortical morphology (Yu et al., 2023). Related morphometric evidence further indicates that internalizing and externalizing comorbidity is associated with distinct patterns of cortical surface area and thickness, with regional differences linked to genetic and cell-type-specific signatures (Kuang et al., 2023). Although these structural findings do not provide a direct explanation for specific TEP components, they emphasize that electrophysiological biomarkers in psychiatric disorders should be interpreted within the anatomical and neurodevelopmental context of the cortex being stimulated.

TEPs have also been applied to evaluate the modulatory effects of antiepileptic drugs on human cortical excitability. Evidence suggests that amplitude changes in early TEP components (<30 ms) following stimulation may reflect the excitability state of the corticospinal system, thereby serving as potential electrophysiological markers of pharmacological effects (Darmani et al., 2019). In stroke research, chronic stroke patients have been reported to exhibit significantly larger P30 amplitudes compared with healthy controls, indicating altered cortical reactivity (Hordacre et al., 2019). Further studies demonstrated not only increased P30 amplitude but also delayed latency in stroke patients, with the degree of P30 latency prolongation correlating with the severity of hand motor impairment (Gray et al., 2017).

TEPs have additionally been widely used to assess the immediate effects of different neuromodulation paradigms on cortical excitation–inhibition balance. For example, studies comparing intermittent theta-burst stimulation (iTBS) and continuous theta-burst stimulation (cTBS) applied to the motor cortex reported increased amplitudes of early TEP components (e.g., P30) following iTBS, consistent with enhanced cortical excitability, whereas cTBS was associated with increased N45 and N100 amplitudes, indicative of augmented inhibitory processes. TEP changes before and after stimulation exhibited good test–retest reliability, supporting their utility as effective markers for monitoring TBS-induced excitation–inhibition dynamics (Ozdemir et al., 2021).

Although these findings support the clinical relevance of TEPs, they should not be interpreted as disorder-specific biomarkers in isolation. Similar changes in TMS-EEG indices of cortical excitability or inhibition can be observed across different neurological and psychiatric conditions. For example, impaired inhibitory responses have been reported in schizophrenia, epilepsy, depression, and autism spectrum disorder, although the affected cortical targets, latency ranges, oscillatory features, and clinical correlates may differ across conditions (Farzan et al., 2010; Gefferie et al., 2023; Santoro et al., 2024; Takano et al., 2025; Mimura et al., 2025). Thus, TEP alterations are better regarded as transdiagnostic indicators of altered cortical reactivity or E/I balance rather than as disease-specific diagnostic signatures. Their clinical interpretation requires integration with stimulation site, symptom profile, disease stage, medication status, and complementary neurophysiological or imaging markers.

#### 2.1.5 Challenges and future directions

Despite the substantial potential of TEPs in both basic and clinical research, their methodological implementation and translational application continue to face several challenges, among which artifact removal remains a central issue. TMS pulses introduce high-amplitude electromagnetic artifacts, scalp muscle contractions, and auditory and somatosensory confounds, which can substantially affect early and mid-latency TEP components. To address these challenges, various artifact-suppression strategies have been proposed and developed, including ICA, SSP, and noise masking. Although these techniques can effectively reduce artifact contamination to some extent, no single approach has proven universally optimal across all experimental conditions.

In practice, combining multiple artifact-handling strategies often further improves signal quality; however, a fundamental trade-off persists between aggressive artifact suppression and preservation of genuine neural signals. Due to the lack of a verifiable “ground truth” for neural responses, the effectiveness of existing artifact-removal methods cannot be directly validated empirically. Consequently, detailed reporting of methodological procedures and cautious interpretation of results are essential when applying different artifact-correction techniques (Table 1).

**Table 1:** Pharmacological interpretation and limitations of canonical TEP components.

TEP component	Common neurophysiological interpretation	Main pharmacological/Experimental evidence	Key limitation
N15/ early components	Early local cortical activation	Direct receptor-level pharmacological evidence remains limited	Highly vulnerable to pulse artifact, muscle activity, and early sensory contamination
P30	Early cortical excitability and local recruitment	Reported in excitability and clinical studies, including AD/MCI and stroke contexts	Less receptor-specific than N45/N100; may reflect mixed excitatory, inhibitory, and state-dependent processes
N45	GABA_A-sensitive inhibitory processing	Alprazolam, diazepam, zolpidem, and S44819 modulate N45; LICI also affects N45	Not purely GABA_A-specific; NMDA receptor antagonism can also increase N45, suggesting broader E/I balance
P60	Intermediate integration and propagated activity	SICI/ICF and rTMS paradigms modulate P60; AMPA receptor antagonism can affect P60	Sensitive to propagation, stimulation parameters, and possible sensory contamination
N100	Slower inhibitory processing, often linked to GABA_B mechanisms	Baclofen and LICI studies support GABA_B involvement	Not purely GABA_B-specific; influenced by auditory/somatosensory input, attention, brain state, and sham-control conditions
P180 / late components	Late network-level and sensory-related processing	Late components are modulated in paired-pulse and pharmacological paradigms	Strong overlap with auditory/somatosensory evoked responses; limited receptor-level specificity

Rather than treating ICA, SSP, or SSP–SIR as universally preferred cleaning strategies, artifact handling in TMS-EEG should be considered in relation to the dominant artifact profile, the latency window of interest, and the expected vulnerability of the study population. ICA is widely used for separating components that are relatively independent from the TMS-evoked neural response, including ocular activity, blinks, and other physiological artifacts that vary across trials (Hernandez-Pavon et al., 2022; Hernandez-Pavon et al., 2023). This approach may be particularly useful when the main concern is residual physiological noise or when mid- and late-latency TEP components are the primary outcomes. However, its application to early time-locked TMS artifacts requires caution. Recent simulation work indicates that when artifact waveforms show limited trial-to-trial variability, ICA-based cleaning may also remove part of the non-artifactual TEP signal, thereby introducing overcorrection rather than simply improving signal quality (Atti et al., 2024). This issue is especially relevant for early TEP components, where neural responses and stimulation-evoked muscle artifacts may overlap closely in time.

For artifacts that are strongly time-locked to the TMS pulse, particularly TMS-evoked muscle activity, SSP-based and source-informed approaches may provide a useful complementary strategy. SSP–SIR was specifically developed to suppress TMS-evoked muscle artifacts by using spatial information to separate artifact-dominated activity from plausible cortical sources (Mutanen et al., 2022; Mutanen et al., 2022). A recent simulation study further suggests that SSP–SIR can outperform ICA in suppressing time-locked muscle artifacts, whereas ICA remains useful for artifacts with greater inter-trial variability, such as ocular activity (Mutanen et al., 2024). Nevertheless, SSP-based approaches should not be regarded as artifact-free solutions. Their performance depends on the spatial separability between artifact and neural topographies, and the signal of interest may still be attenuated during correction (Mutanen et al., 2022; Mutanen et al., 2024). Therefore, in studies focusing on early cortical reactivity, especially when stimulation is delivered near cranial muscles or when patient groups show high artifact burden, SSP–SIR or related source-informed approaches may be considered alongside ICA-based cleaning, with sensitivity analyses used to evaluate the robustness of key findings.

This consideration is particularly important for clinical TMS-EEG studies, where artifact profiles may differ substantially from those observed in healthy controls. In healthy participants with stable recording conditions and relatively low artifact burden, ICA-based pipelines combined with pulse-artifact interpolation, bad-trial rejection, and sensory control may be sufficient for many conventional TEP analyses (Rogasch et al., 2017; Hernandez-Pavon et al., 2023). By contrast, in patient cohorts with involuntary muscle activity, reduced tolerance to stimulation, excessive blinking or movement, medication effects, or altered cortical anatomy, preprocessing choices should be more explicitly justified and validated. For example, in stroke studies targeting early motor cortical responses, source-informed artifact suppression may be helpful when stereotyped muscle artifacts dominate the early post-stimulation interval, but its assumptions should be considered carefully in the presence of lesions, cortical reorganization, or unavailable individualized head models. Conversely, when late components such as N100 or P180 are the main outcomes, preprocessing should be combined with optimized auditory and somatosensory control, because these components are especially vulnerable to sensory-evoked contributions. Thus, the choice between ICA, SSP, and SSP–SIR should be framed as a context-dependent methodological decision rather than a fixed hierarchy of preprocessing methods. These considerations are summarized in Table 2, which outlines practical but non-prescriptive criteria for selecting artifact-removal strategies according to artifact characteristics, latency range, and clinical context.

**Table 2:** Context-dependent considerations for artifact removal in TMS-EEG.

Analytical context	Main concern	Methodological consideration
Ocular, blink, cardiac, or other physiological artifacts with trial-to-trial variability	These artifacts may be separable from TMS-evoked neural activity	ICA is commonly used, but component rejection should be based on time course, topography, and spectrum rather than automatic classification alone.
Early TEP components, especially within the first 50 ms	Neural responses may overlap with TMS-evoked muscle artifacts	ICA should be used cautiously; SSP–SIR or source-informed approaches may be considered when muscle artifacts are strongly time-locked to the TMS pulse.
Stimulation sites close to cranial muscles, including M1, lateral frontal, or temporal regions	Early muscle artifacts may dominate the post-stimulation signal	Acquisition-level reduction of muscle activation remains essential; source-informed artifact suppression may complement, but not replace, careful setup.
Healthy participants with stable recordings and low artifact burden	Residual physiological artifacts are usually manageable	A conventional pipeline combining pulse-artifact interpolation, bad-trial rejection, ICA, and sensory control may be sufficient for many TEP analyses.
Clinical cohorts with movement, poor tolerance, medication effects, lesions, or altered anatomy	Artifact structure and neural topographies may differ from healthy controls	Pipeline choices should be justified, and key findings should preferably be tested with sensitivity analyses across reasonable preprocessing options.
Biomarker or treatment-response studies	Preprocessing differences may affect group comparisons or prediction models	The same predefined pipeline should be applied across groups, with transparent reporting of rejected trials, removed components, and retained signal quality.

Methodological variability, including differences in stimulation parameters (e.g., intensity and frequency), coil type, participant setup, and data acquisition and processing pipelines, can lead to substantial inconsistencies in TEP findings across studies. Importantly, some of these sources of variability arise before analysis begins, underscoring the value of careful data collection practices and transparent reporting at the acquisition stage. Beck, Heyl et al. (2024) systematically highlighted unresolved issues in device configuration, experimental design, and computational workflows within the current TMS-EEG literature, emphasizing that such methodological heterogeneity significantly constrains reproducibility and inter-laboratory comparability. Greater consistency in experimental design, acquisition procedures, and analysis choices may help mitigate these sources of variability and improve the interpretability and reliability of TMS-EEG research.

Building on these considerations, future TEP studies should prioritize harmonization through transparent and reproducible reporting rather than through a single mandatory preprocessing pipeline (Tremblay et al., 2019; Hernandez-Pavon et al., 2023). At the acquisition level, studies should explicitly report stimulation intensity calibration, coil position and orientation, neuronavigation procedures, pulse waveform, the number of delivered and retained trials, auditory and somatosensory control procedures, and participant state during recording (Hernandez-Pavon et al., 2023; Beck, Heyl et al., 2024). At the preprocessing level, key parameters should include artifact-rejection criteria, interpolation windows around the TMS pulse, filtering settings, bad-channel and bad-trial handling, re-referencing strategy, and the implementation details of ICA, SSP, or other artifact-suppression procedures (Rogasch et al., 2017; Hernandez-Pavon et al., 2023; Brancaccio et al., 2024). At the component-analysis level, reports should specify the predefined latency windows, electrode selections, peak or mean amplitude definitions, and statistical correction methods used for TEP quantification (Tremblay et al., 2019; Hernandez-Pavon et al., 2023). Rather than prescribing a universally optimal cleaning strategy, such reporting would make pipeline choices more transparent and reproducible, particularly because different preprocessing approaches may introduce systematic differences in TEP amplitude, spatiotemporal pattern, and inter-trial variability (Rogasch et al., 2017; Brancaccio et al., 2024).

Furthermore, inter-individual differences in cortical anatomy, transient brain states (e.g., fatigue, vigilance, and attentional level), and medication use can all influence TEP amplitude and latency. If not adequately accounted for in experimental design or statistical modeling, these factors may constitute major sources of unexplained variability. Future studies should therefore account more systematically for individual-level factors in both study design and data interpretation (Tremblay et al., 2019).

Although the biomarker potential of TEPs has been preliminarily supported by multiple studies, their clinical utility has not yet been firmly established. Most existing studies are limited by small sample sizes and predominantly descriptive designs, with a lack of large-scale randomized controlled trials (RCTs) to rigorously validate their diagnostic and prognostic value. A narrative review by Cruciani et al. (2023) highlighted the promising role of TMS-EEG in evaluating neuromodulatory interventions such as rTMS, while emphasizing that clinical translation will require high-quality RCTs to confirm the reliability of TEPs as diagnostic indicators (e.g., cortical excitability in depression) and predictors of treatment response.

Overall, current TEP research remains largely descriptive and may be influenced by sample homogeneity, methodological bias, and insufficient cross-study comparability. Future progress will depend on improved artifact handling, transparent and reproducible reporting practices, replication of established protocols, and large-sample validation, in order to facilitate the transition of TEPs from mechanistic descriptors toward clinically actionable biomarkers with predictive value.

### 2.2 Global and local mean field power

#### 2.2.1 Definition

Although TEP components at different latencies can capture the temporal evolution of excitation and inhibition following TMS, such analyses often rely on specific electrodes or individual components, which may limit a comprehensive characterization of the spatial distribution of TMS-evoked responses. To address this limitation, spatially integrated amplitude metrics based on multichannel EEG signals have been introduced.

**Figure 2:**
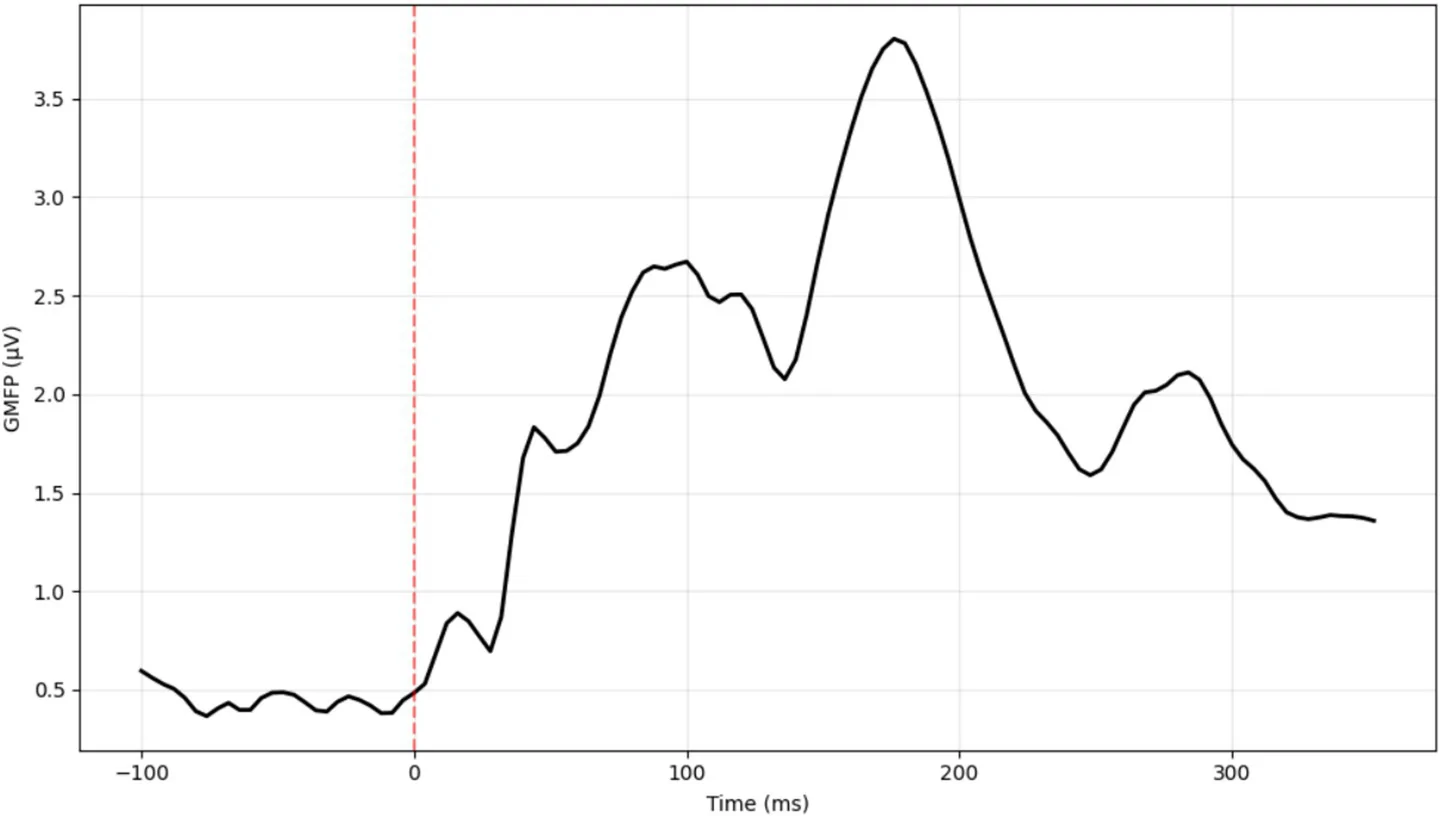
Schematic illustration of global mean field power. The figure illustrates the conceptual time course of GMFP calculated from multichannel EEG responses following TMS. The vertical dashed line indicates the timing of the TMS pulse. This figure is provided for illustrative purposes only and does not present condition-wise or group-wise experimental data. Original schematic illustration created by the authors for this review.

GMFP integrates EEG activity across the entire scalp to quantify the overall strength of TMS-evoked responses at the whole-brain level. In contrast, LMFP is computed within predefined clusters of electrodes and is used to assess response magnitude and excitability changes within specific cortical regions (Romero Lauro et al., 2014). To clarify the relationship between multichannel TEP waveforms and spatially integrated response measures, Figure 2 provides a schematic illustration of the GMFP time course derived from scalp-level EEG signals. This figure is intended to show how GMFP summarizes the global magnitude of TMS-evoked activity across electrodes rather than to present condition-wise experimental results.

Because both GMFP and LMFP are derived from the statistical properties of multichannel signals, these metrics typically exhibit a relatively high signal-to-noise ratio and reduced sensitivity to noise or sporadic artifacts affecting individual electrodes. As such, they are considered important complements to conventional single-channel or single-component TEP analyses. GMFP primarily captures the global pattern of TMS-evoked activity, whereas LMFP provides greater sensitivity to spatially specific changes in local cortical excitability (Casula et al., 2018).

#### 2.2.2 Characteristics and temporal components

The temporal dynamics of GMFP and LMFP are commonly characterized by peak amplitudes within specific post-stimulation time windows. Based on latency, these responses are typically divided into early (0–50 ms), middle (50–100 ms), and late (100–150 ms) components, corresponding to immediate local cortical activation, secondary network feedback, and slower long-range inhibitory or integrative processes, respectively (Varoli et al., 2018).

Following stimulation of the M1, GMFP often exhibits a rapid and pronounced increase in the early time window, which is generally attributed to synchronous firing of pyramidal neurons and local cortical depolarization. In contrast, LMFP shows more prominent peaks in electrode clusters proximal to the stimulation target (e.g., M1 or DLPFC), with amplitudes that are highly sensitive to stimulation intensity, coil orientation, and pulse waveform. For example, monophasic pulses delivered in a posterior–anterior (PA) direction typically elicit higher LMFP peaks, suggesting enhanced local cortical excitability (Casula et al., 2018).

Further evidence indicates that GMFP peaks may persist for up to 200 ms or longer following stimulation, reflecting the propagation and integration of TMS effects across large-scale functional networks. In clinical conditions such as stroke, this propagation may be influenced by hemispheric asymmetry, transcallosal interactions, and the integrity of residual motor pathways, consistent with broader evidence that hemispheric asymmetry and callosal organization shape interhemispheric communication (Wu et al., 2025). Thus, GMFP and LMFP may help characterize not only the magnitude of TMS-evoked responses, but also their redistribution across ipsilesional and contralesional networks. By comparison, LMFP is more sensitive to local parameter changes. For instance, rotating the coil orientation from 0° (posterior) to 90° (lateral) can significantly enhance early LMFP components, highlighting the critical role of stimulation orientation in shaping local cortical recruitment patterns (Casula et al., 2022).

In addition to stimulation parameters, these spatially integrated metrics are modulated by individual factors. Studies in middle-aged and older adults have shown that LMFP amplitude following DLPFC stimulation is positively correlated with working memory and reasoning performance, suggesting potential biomarker value for cognitive function assessment (Redondo-Camós et al., 2022). It should be noted that early GMFP components may be amplified by non-neural artifacts such as muscle activity; however, with appropriate experimental design and preprocessing procedures, such influences can be mitigated to a considerable extent (Bertazzoli et al., 2021).

#### 2.2.3 Data analysis methods

The computation of GMFP and LMFP is typically based on standardized TMS-EEG data-processing pipelines, but their reliability is also influenced by data collection conditions such as trial number, stimulation stability, and recording quality. First, TEP signals from repeated trials are averaged (generally 50–200 trials) to enhance signal-to-noise ratio and reduce the impact of random noise. Subsequently, mean field power is calculated within the time window of interest based on voltage values across EEG channels.

The mathematical definition of GMFP is given by:


$$\text{GMFP}(t)=\sqrt{\frac{1}{M}\sum_{j=1}^{M}{\left[v_{j}(t)-\bar{v}(t)\right]}^{2}}$$

where M denotes the total number of EEG channels, $v_{j}(t)$ represents the voltage (in μV ) at time t for the j -th channel, and $\bar{v}(t)$ is the mean voltage across all channels at that time point.

The computation of LMFP follows the same principle as GMFP but is restricted to a predefined local electrode cluster, typically comprising 4–6 electrodes over frontal or parietal regions, thereby increasing spatial specificity for the assessment of cortical responses within targeted areas (Romero Lauro et al., 2014).

#### 2.2.4 Clinical and research applications

As spatially integrated complements to conventional TEP measures, GMFP and LMFP have demonstrated substantial potential in the evaluation of neuromodulatory effects and in mechanistic studies of neuropsychiatric disorders.

In depression research, continuous theta-burst stimulation (cTBS) applied to the DLPFC has been shown to induce a significant reduction in LMFP, reflecting enhanced local inhibitory plasticity. Importantly, this LMFP modulation has been associated with the prediction of subsequent rTMS treatment response, supporting its potential utility as a neurophysiological marker of treatment sensitivity (Rocchi et al., 2018). In Alzheimer’s disease, elevated LMFP has been observed in the precuneus, with amplitude increases correlating with the severity of cognitive decline. These findings support LMFP as a potential biomarker of abnormal cortical excitability in AD, particularly when interpreted in combination with TEP measures, which together provide enhanced explanatory power (Casula et al., 2023).

In stroke research, GMFP and LMFP are particularly relevant because motor recovery is closely linked to the reorganization of ipsilesional and contralesional networks rather than to local cortical excitability alone. TMS-EEG studies have shown that stroke patients may exhibit abnormal interhemispheric dynamics, including reduced inhibition from the affected to the unaffected hemisphere and relatively preserved inhibition from the unaffected to the affected hemisphere, resulting in an interhemispheric imbalance that is associated with hand motor recovery and callosal microstructural integrity (Casula et al., 2021). More recent evidence further suggests that TMS-EEG-derived interhemispheric balance indices and low-frequency power in the affected hemisphere are associated with upper limb impairment in chronic stroke, supporting the use of TMS-EEG to characterize individualized recovery-related network states (Martino Cinnera et al., 2025). From a broader network perspective, dynamic imaging studies indicate that in moderate-to-severe subcortical stroke, strengthened interactions between the contralesional dorsal premotor cortex and bilateral subcortical networks may serve as a compensatory pathway for motor recovery (Fan et al., 2026). These findings suggest that LMFP or GMFP changes after stimulation should be interpreted in relation to hemispheric asymmetry, lesion severity, and compensatory recruitment of premotor and subcortical pathways, rather than as simple markers of local response amplitude.

Within neuromodulation paradigms, GMFP and LMFP provide quantitative tools for assessing the impact of stimulation parameters on cortical responses. For example, cathodal transcranial direct current stimulation (tDCS) applied to the parietal cortex did not induce significant changes in either GMFP or LMFP, suggesting limited inhibitory effects on higher-order cortical regions under these conditions (Varoli et al., 2018). In contrast, studies employing controllable TMS (cTMS) have demonstrated that longer pulse widths (>100 μs) significantly increase LMFP amplitude, offering a rationale for the optimization of individualized stimulation parameters (Casula et al., 2018).

Furthermore, reliability assessments of theta-burst stimulation (TBS) effects indicate that LMFP exhibits moderate test–retest consistency in detecting DLPFC excitability changes, with concordance correlation coefficients (CCC) in the range of approximately 0.4–0.6. These findings highlight the potential utility of LMFP in longitudinal follow-up and intervention monitoring, while also underscoring the need for further improvement in measurement stability (Moffa et al., 2022).

#### 2.2.5 Challenges and future directions

Despite the demonstrated value of GMFP and LMFP in TMS-EEG research, several methodological and translational challenges remain. Different artifact-removal strategies (e.g., ICA versus SSP) can substantially influence GMFP/LMFP amplitude characteristics and spatial distributions, thereby limiting comparability across studies (Bertazzoli et al., 2021). In neuromodulatory paradigms such as TBS, the between-session reliability of LMFP in detecting stimulation effects remains limited (CCC < 0.5), potentially due to residual artifacts, variability in stimulation targeting, or fluctuations in participant brain state (Moffa et al., 2022).

Variability in pulse parameters, including pulse width, pulse waveform, and coil orientation, as well as differences in stimulation delivery and the definition of local electrode clusters, can further amplify inter-individual differences. These factors highlight the need for clearer reporting of both acquisition conditions and analytical choices, as well as more consistent interpretive practices across studies (Casula et al., 2018; Casula et al., 2022; Hernandez-Pavon et al., 2023).

Building on these issues, standardization of GMFP and LMFP should focus on the *a priori* definition and transparent reporting of electrode sets, time windows, amplitude metrics, and preprocessing dependencies, rather than on imposing a single universal analysis pipeline. Because LMFP is computed within local regions of interest, it is particularly sensitive to the choice of local electrode clusters. Future studies should therefore avoid *post hoc* cluster selection and should clearly state whether clusters were defined anatomically, centered on the stimulation target, or adopted from previous literature (Romero Lauro et al., 2014; Cruciani et al., 2023). Reports should also specify whether GMFP was calculated across the full scalp or a specific electrode montage, whether LMFP clusters comprised contiguous electrodes or source-informed regions, and whether amplitudes were averaged within predefined time windows or extracted as peak values. In addition, GMFP/LMFP findings should be interpreted together with TEP waveforms, scalp topographies, and, where available, source-space estimates or MRI-guided targeting information, rather than being treated as isolated global amplitude markers (Michel and Brunet, 2019; Hernandez-Pavon et al., 2023). Such practices would improve cross-study comparability and help determine whether observed changes reflect genuine local or network-level cortical reactivity, residual artifacts, or differences in analytical definitions.

Although GMFP and LMFP show promise as predictive biomarkers, particularly in higher-order cognitive cortical regions, their clinical utility requires validation through larger-scale randomized controlled trials and longitudinal studies (Redondo-Camós et al., 2022). In this regard, future research should not only examine whether GMFP/LMFP can detect group-level differences or intervention-induced changes, but also determine whether these metrics provide stable, individually meaningful information that can support prognosis, treatment stratification, or monitoring of neuromodulatory effects.

Overall, GMFP and LMFP provide a spatially integrated perspective on TMS-evoked cortical responses that extends beyond single TEP components. However, their stable and clinically meaningful application will depend on clearer cross-study comparability, predefined spatial and temporal analysis strategies, careful interpretation of individual variability, multimodal support for physiological interpretation, and the accumulation of high-quality longitudinal evidence.

## 3 Time-frequency analysis

### 3.1 Definition

Building upon time-domain TEP component analysis and spatially integrated measures (GMFP/LMFP), time-frequency analysis further characterizes TMS-evoked EEG activity along the frequency dimension. Because TMS-EEG signals are inherently non-stationary, analyses relying solely on time-domain or frequency-domain approaches are often insufficient to capture the rapid and transient changes in cortical oscillatory activity induced by stimulation. Time-frequency analysis therefore constitutes an essential component of TMS-EEG data processing, enabling the joint characterization of post-stimulation dynamics in both time and frequency domains.

Time-frequency analysis allows simultaneous assessment of power modulation and phase dynamics following TMS. Commonly used metrics include TRSP and ITPC. TRSP primarily reflects increases or decreases in oscillatory power, whereas ITPC quantifies the degree of phase locking across trials (Pigorini et al., 2011; Manganotti et al., 2012). Compared with analyses focused on discrete TEP components at specific latencies, time-frequency approaches provide a more continuous and multidimensional description of TMS-induced network state transitions and short-term plasticity.

TRSP is the most widely used time-frequency metric in TMS-EEG and quantifies the trial-averaged induced time-frequency power. It incorporates both phase-locked and non–phase-locked components and can be considered an extension of traditional event-related spectral perturbation (ERSP) methods adapted for TMS paradigms. After baseline normalization, positive and negative TRSP changes correspond to power enhancement (ERS-like) and power suppression (ERD-like), respectively, thereby reflecting the temporal–spectral dynamics of TMS-induced oscillations. TRSP is typically computed as:


$$\text{TRSP}(f,t)=\frac{1}{n}\sum_{k=1}^{n}{|F_{k}(f,t)|}^{2}$$

Where $F_{k}(f,t)$ denotes the complex time-frequency estimate at frequency f and time t for the k -th trial, and n represents the total number of trials.

In contrast to TRSP, which focuses on oscillatory power, ITPC characterizes cross-trial consistency of phase alignment in TMS-evoked responses. ITPC quantifies whether instantaneous phases across trials are stably locked at a given time-frequency point. Values approaching 1 indicate strong phase locking, whereas values near 0 indicate random phase distributions:


$$\text{ITPC}(f,t)=\frac{1}{n}\sum_{k=1}^{n}\frac{F_{k}(f,t)}{|F_{k}(f,t)|}$$

Where $F_{k}(f,t)/|F_{k}(f,t)|$ represents the unit phase vector for the *k*-th trial, and the magnitude of the averaged vector reflects phase consistency across trials.

Overall, by jointly characterizing power modulation (TRSP) and phase synchronization (ITPC), time-frequency analysis provides a multidimensional perspective on rapid oscillatory modulation induced by TMS. This approach allows investigation of dynamic network processes beyond static power distributions and is particularly well suited for capturing short-term plasticity and state-dependent effects elicited by TMS.

### 3.2 Characteristics and oscillatory features

Time-frequency metrics are highly sensitive to stimulation parameters and ongoing brain state, exhibiting pronounced differences across frequency bands and temporal windows. Compared with purely time-domain measures, TRSP and ITPC reveal transient oscillatory dynamics induced by TMS, offering richer insights into the mechanisms underlying cortical responses.

Following stimulation of the M1, TRSP often shows early (<100 ms) increases in β–γ band power, which are commonly interpreted as reflecting rapid network resetting and synchronization processes (Saari et al., 2018). These findings suggest that TMS can reorganize local and connected network oscillatory states on very short time scales. In contrast, ITPC primarily captures cross-trial phase locking; for example, in memory or attention task contexts, enhanced θ-band ITPC indicates stronger alignment of TMS-evoked activity with intrinsic neural rhythms, reflecting modulation of task-relevant network states (Beck et al., 2024).

State dependency is another important feature of TMS-induced oscillatory responses. Pre-stimulus oscillatory power can shape the magnitude of subsequent TMS-evoked activity, indicating that TRSP and ITPC should be interpreted not only as post-stimulation responses, but also in relation to the ongoing neural state before stimulation (Farzan et al., 2016; Poorganji et al., 2023). This issue is particularly relevant for clinical translation and is discussed in detail in Section 3.5.

Within the TMS-EEG framework, time-frequency analysis has been widely applied to investigate dynamic regulation of E/I balance. In general, power enhancement in higher-frequency TRSP, especially within the β (13–30 Hz) and γ (>30 Hz) bands, is often considered indicative of excitatory processes, reflecting glutamate-mediated local neuronal synchronization and integrative activation. For instance, early TMS-induced γ-band bursts have been closely linked to glutamatergic-mediated local excitation (Belardinelli et al., 2021).

Conversely, changes in lower-frequency oscillations are more frequently associated with inhibitory modulation. *α*-band (8–12 Hz) desynchronization (ERD-like) and θ-band (4–8 Hz) synchronization or power modulation are commonly interpreted as reflecting GABA_A- or GABA_B- mediated network resetting and inhibitory control. Pharmacological studies provide direct support for this interpretation: GABA_A agonists such as diazepam and zolpidem significantly modulate α/β oscillations, with associated power changes considered indirect markers of enhanced inhibition (Premoli et al., 2014).

Further time-frequency investigations indicate that long-interval intracortical inhibition (LICI)-related suppressive effects are not confined to a single frequency band or local cortical site but may extend across *θ*–β bands and spatially involve distant brain regions. These findings suggest that E/I imbalance may arise from impaired network-level inhibitory regulation rather than dysfunction of isolated cortical nodes (Takano et al., 2025; Mimura et al., 2025). It should be emphasized that scalp-level time-frequency results alone may underestimate spatial specificity; therefore, interpretations of E/I mechanisms should be informed by β-band modulation patterns and source-level analyses to avoid oversimplified physiological inferences (Garcia Dominguez et al., 2014).

### 3.3 Data analysis methods

Time-frequency analysis in TMS-EEG is commonly based on wavelet transform (WT), short-time Fourier transform (STFT), or Hilbert–Huang transform (HHT), with HHT demonstrating particular advantages in detecting the onset timing of TMS-induced oscillations (Pigorini et al., 2011). Standard pipelines typically include averaging 50–200 trials to enhance signal-to-noise ratio, artifact removal using methods such as ICA, and computation of TRSP and ITPC within a predefined post-stimulation window (commonly 0–500 ms) (Manganotti et al., 2012). Because high-frequency time-frequency estimates are especially vulnerable to muscle and coil-related contamination, careful data collection and stable recording conditions are particularly important at the acquisition stage.

High-density EEG and neuronavigation systems improve spatial precision; however, different time-frequency transformation methods may still introduce approximately 20% variability in results (Bertazzoli et al., 2021). For studies targeting E/I mechanisms, some approaches further incorporate source estimation techniques (e.g., sLORETA) to project time-frequency power onto cortical source space, enabling quantification of GABA_B-mediated inhibitory effects (Mimura et al., 2025). Statistical analyses typically rely on permutation testing with appropriate correction for multiple comparisons to ensure robustness (Premoli et al., 2014).

### 3.4 Clinical and research applications

Time-frequency metrics have demonstrated substantial value in elucidating neuromodulatory effects and disease-related network mechanisms. In stroke research, increased β-band TRSP following iTBS intervention has been shown to correlate significantly with motor function recovery, suggesting that β oscillations contribute to plastic reorganization of motor networks (Ding et al., 2022). This interpretation is strengthened by recent TMS-EEG evidence showing that contralesional M1 can modulate ipsilesional M1 β oscillatory activity after acute ischemic stroke, indicating that post-stroke β-band responses may reflect not only local cortical reactivity but also altered interactions between the two motor cortices (Jia et al., 2025). These findings suggest that time-frequency measures may be particularly useful for characterizing recovery-related changes in sensorimotor network dynamics. In depression, baseline *θ*-band ITPC has been reported to predict clinical response to rTMS, indicating that phase synchronization characteristics may index individual susceptibility to neuromodulation (Hasanzadeh et al., 2019). In pain-related studies, reduced *α*-band TRSP following M1 stimulation has been interpreted as reflecting dynamic regulation of sensorimotor networks, providing a temporally resolved electrophysiological marker of cortical alterations associated with pain states (De Martino et al., 2024).

Within neuromodulation paradigms, state-locked TMS has been shown to markedly enhance ITPC, supporting the use of time-frequency metrics for parameter optimization and real-time feedback in closed-loop stimulation systems (Bai et al., 2023; Wang et al., 2024). In tinnitus patients, reductions in *γ*-band TRSP following rTMS have been correlated with symptom alleviation, suggesting that suppression of abnormal high-frequency oscillations may underlie therapeutic effects (Schecklmann et al., 2015).

Regarding assessment of E/I balance, time-frequency analysis offers distinct advantages across multiple neuropsychiatric conditions. In treatment-resistant depression (TRD), time-frequency analysis of LICI over the DLPFC has revealed attenuated θ–β band power suppression, indicating impaired GABA_B -mediated inhibitory function and supporting its role as a potential pathophysiological marker (Takano et al., 2025). Pharmacological studies further demonstrate that zolpidem significantly modulates TMS-induced α-band oscillatory changes, supporting the use of TRSP metrics to assess enhancement of inhibitory neurotransmission (Premoli et al., 2014). In autism spectrum disorder (ASD), source-resolved time-frequency analyses have revealed reduced LICI-related inhibitory effects closely linked to E/I imbalance, providing a potential basis for individualized rTMS intervention strategies (Mimura et al., 2025; Casanova et al., 2020). Additionally, in cerebello–cortical connectivity studies, enhanced high-β TRSP has been used to assess pathway integrity during post-stroke functional recovery (Gassmann et al., 2022).

However, the presence of attenuated LICI-related suppression or abnormal oscillatory responses across different disorders also underscores a key translational limitation: time-frequency measures may be sensitive to altered inhibitory network regulation, but they are not necessarily specific to a single diagnostic category. Despite the promising role of time-frequency analysis in individualized treatment optimization, most existing studies employ relatively small sample sizes, and the generalizability of findings requires validation through multicenter investigations.

### 3.5 State-dependency and baseline oscillatory activity in clinical translation

TMS-EEG responses are intrinsically state-dependent. A TMS pulse does not act on a physiologically neutral cortex, but interacts with ongoing neural activity at the time of stimulation. Accordingly, the amplitude and spectral profile of TEPs and TRSP may be shaped by the oscillatory state immediately preceding the pulse. Recent TMS-EEG evidence suggests that pre-stimulus power, rather than phase alone, can predict the magnitude of subsequent cortical responses, supporting the view that TMS-evoked activity reflects an interaction between external stimulation and endogenous brain dynamics (Farzan et al., 2016; Poorganji et al., 2023). This point is particularly relevant for clinical studies, where baseline EEG activity is often altered by disease-related pathophysiology.

In Alzheimer’s disease, resting-state EEG commonly shows a slowing pattern, characterized by increased delta/theta activity, reduced alpha/beta activity, and slowing of the peak alpha frequency (Cassani et al., 2018; Kopčanová et al., 2024). After stroke, EEG abnormalities may include reduced higher-frequency activity over the lesioned hemisphere and altered interhemispheric balance between affected and unaffected networks (Keser et al., 2022). Such baseline abnormalities can complicate the interpretation of TMS-EEG findings. For example, a group difference in P30, N45, or N100 amplitude may reflect altered cortical reactivity, but it may also be influenced by differences in vigilance, medication status, fatigue, or resting oscillatory activity. Similarly, TRSP estimates are especially sensitive to baseline state because post-stimulation power is usually expressed relative to a pre-stimulus interval. In a group with elevated theta power or reduced beta power at baseline, relative power changes may therefore partly reflect the scaling effect of baseline normalization rather than a purely TMS-induced modulation (Grandchamp and Delorme, 2011).

For clinical translation, baseline oscillatory activity should therefore be treated as part of the physiological context in which TMS-EEG responses are generated. In addition to reporting TEPs and TRSP, studies should describe baseline spectral power in the relevant frequency bands, electrodes, or source regions. When clinical and control groups differ in baseline power, statistical models should account for these differences rather than relying only on percentage change or relative normalization. Including baseline power as a covariate in ANCOVA or regression models can help separate disease-related baseline abnormalities from stimulation-evoked responses, especially because percentage change from baseline may be sensitive to baseline variance (Vickers, 2001). For trial-level analyses, mixed-effects models may further allow pre-stimulus power, group, stimulation condition, and their interactions to be modeled within the same framework.

This consideration is also important in longitudinal and treatment-response studies. Changes in TEP or TRSP after an intervention may indicate altered cortical reactivity, but they may also reflect shifts in baseline oscillatory state across sessions. Recording resting-state EEG before TMS, monitoring vigilance and fatigue, documenting medication status, and applying comparable recording conditions across groups and sessions would therefore improve interpretability. Rather than treating baseline oscillatory differences only as nuisance variance, future clinical TMS-EEG studies may use them to refine the interpretation of evoked responses and to distinguish altered cortical reactivity from altered brain state.

### 3.6 Integrating multidimensional TMS-EEG responses

TEPs, GMFP/LMFP, and time-frequency measures are often analyzed separately, but they describe different aspects of the same TMS-evoked response. TEPs capture phase-locked voltage deflections after stimulation, GMFP and LMFP summarize the global or local strength of these multichannel responses, and TRSP/ITPC describe how the response is expressed through changes in oscillatory power and phase consistency. Together, these measures provide temporal, spatial, and spectral views of cortical reactivity.

This relationship is better understood as convergence across analytical domains rather than as a fixed mapping between individual peaks and frequency bands. For example, an increase in local P30 amplitude may suggest enhanced early cortical reactivity, particularly if it is accompanied by increased LMFP over the stimulated region. If early TRSP or ITPC changes are also observed, they may indicate that this response is associated with stronger oscillatory power modulation or phase alignment. However, a larger P30 should not be assumed to correspond to a specific theta-band synchronization event. The dominant oscillatory pattern depends on stimulation site, baseline brain state, stimulation parameters, and the network properties of the targeted region (Rosanova et al., 2009; Pigorini et al., 2011; Pellicciari et al., 2017).

The distinction between evoked and induced activity is also important. A prominent TEP component may coincide with increased ITPC, reflecting stronger phase alignment across trials, without necessarily producing a large TRSP change. Conversely, TRSP changes may occur with little change in the averaged TEP waveform when the oscillatory response is not phase-locked across trials. Interpreting TEPs together with TRSP and ITPC can therefore help clarify whether an effect is mainly expressed as evoked voltage amplitude, phase consistency, oscillatory power, or a combination of these features (Pigorini et al., 2011; Manganotti et al., 2012).

GMFP and LMFP provide an additional spatial anchor for this interpretation. A focal TEP change accompanied by increased LMFP but limited GMFP change may indicate a predominantly local response, whereas broader GMFP enhancement with distributed TRSP or ITPC changes may suggest propagation through connected cortical networks. This view is consistent with evidence that TMS-evoked oscillations reflect both local cortical properties and rapid interactions with distant regions, and that different cortical areas may show preferred oscillatory responses rather than a uniform frequency profile (Rosanova et al., 2009; Romero Lauro et al., 2014).

**Figure 3:**
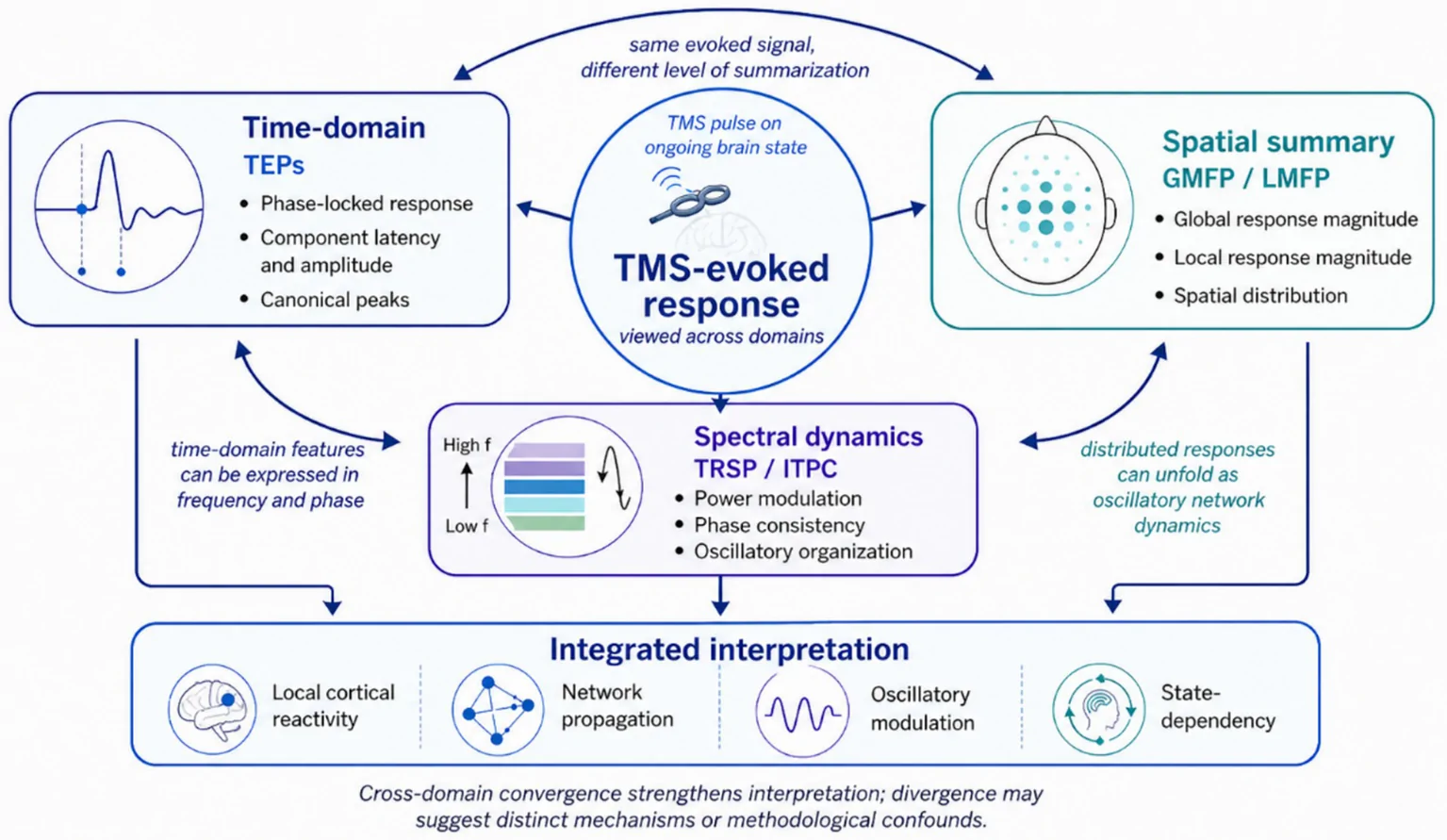
A multidimensional framework for interpreting TMS-EEG responses. TEPs, GMFP/LMFP, and TRSP/ITPC represent complementary analytical views of the same TMS-evoked cortical response. TEPs describe time-domain waveform features, GMFP/LMFP summarize global and local spatial response magnitude, and TRSP/ITPC characterize spectral dynamics through power modulation and phase consistency. Integrating these measures may provide a more coherent interpretation of local reactivity, network propagation, oscillatory modulation, and state-dependency. Cross-domain convergence supports interpretation, whereas divergence may suggest distinct mechanisms or methodological confounds.

For clinical studies, this integrated interpretation may help avoid overreliance on isolated metrics. An altered P30 in Alzheimer’s disease or stroke, for instance, would be more informative when considered together with local LMFP, baseline spectral power, and early TRSP/ITPC changes. Similarly, late components such as N100 may gain interpretive value when examined alongside GMFP/LMFP topography and time-frequency features, while still accounting for sensory co-activation and baseline state. The aim is not to assign each TEP component to a single oscillatory band, but to determine whether time-domain, spatial, and spectral measures support the same physiological interpretation or reveal distinct aspects of the response. Figure 3 illustrates this relationship by placing TEP components, field-power measures, and oscillatory modulation along a shared temporal axis. It is intended as a conceptual guide, not as a deterministic mapping between TEP peaks and frequency bands.

### 3.7 Challenges and future directions

Although time-frequency analysis provides critical insights into dynamic neural modulation induced by TMS, several challenges remain. First, time-frequency metrics are highly sensitive to artifacts, particularly electromyographic activity and coil-related high-frequency noise, which can substantially distort *γ*- and high-*β*-band TRSP and lead to misinterpretation of excitatory or inhibitory effects (Premoli et al., 2014; Muthukumaraswamy, 2013; Hipp and Siegel, 2013). This issue is especially important for high-frequency TMS-EEG analyses, because residual muscle activity may overlap spectrally with β- and γ-band neural activity and can be difficult to fully separate from genuine oscillatory responses.

Second, heterogeneity in signal-processing strategies, including the choice of time-frequency transform, epoch length, baseline correction or normalization methods, trial-rejection criteria, and artifact-removal pipelines, can significantly affect the reproducibility and comparability of TRSP and ITPC results across studies (Beck et al., 2024; Hernandez-Pavon et al., 2023; Pernet et al., 2020). Unlike conventional averaged TEP analysis, time-frequency analysis often requires single-trial decomposition before averaging in order to preserve induced oscillatory activity that is related to, but not necessarily phase-locked to, the TMS pulse. Therefore, differences in trial-level preprocessing, interpolation around the TMS pulse, baseline handling, and normalization strategy may introduce systematic variability in the estimated magnitude and temporal profile of oscillatory responses (Hernandez-Pavon et al., 2023; Brancaccio et al., 2024).

Furthermore, most current studies still focus primarily on band-specific power changes or phase-locking indices, whereas higher-order dynamical features such as cross-frequency coupling are less frequently examined. This may underestimate the complexity of network-level modulation induced by TMS, particularly when time-frequency metrics are used to infer excitation–inhibition balance or state-dependent plasticity (Wang et al., 2024). In assessments of E/I balance, although GABAergic modulation is theoretically specific, its electrophysiological representation remains susceptible to non-neural noise, especially in high-frequency oscillatory analyses.

To address these challenges, future studies should adopt more standardized and transparent reporting of TRSP and ITPC analyses. For TRSP, reports should specify the time-frequency decomposition method, frequency range, sub-band definitions, number of cycles or spectral smoothing parameters, baseline interval, normalization procedure, and whether power estimates were averaged across trials before or after baseline correction. For ITPC, studies should clearly report the phase-estimation method, trial-inclusion criteria, number of retained trials, frequency and time windows of interest, and statistical correction procedures. In addition, reports should describe artifact-rejection strategies, interpolation windows around the TMS pulse, handling of residual muscle activity, and correction for multiple comparisons across time, frequency, and electrodes (Hernandez-Pavon et al., 2023; Pernet et al., 2020). Particular caution is required when interpreting high-β and γ-band effects, because these frequency ranges are especially vulnerable to residual cranial muscle activity and coil-related high-frequency artifacts (Muthukumaraswamy, 2013; Hipp and Siegel, 2013). More transparent reporting of time-frequency parameters would help determine whether observed oscillatory differences reflect genuine TMS-induced neural modulation or methodological heterogeneity.

Future research should also integrate time-frequency metrics more closely with TEPs, GMFP/LMFP, source-space estimates, and multimodal imaging information. Such integration may help determine whether band-specific TRSP or ITPC changes correspond to local cortical reactivity, distributed network propagation, or broader state-dependent modulation. Development of real-time or near real-time time-frequency algorithms will further facilitate implementation of closed-loop TMS systems. Integration of artificial intelligence and automated analysis tools may ultimately enable a transition of TMS-EEG time-frequency metrics from descriptive characterization toward predictive and intervention-oriented applications, thereby accelerating clinical translation.

## 4 Discussion and conclusion

TMS-EEG metric, including TEPs, spatially integrated measures such as GMFP/LMFP, and time-frequency indices such as TRSP and ITPC, provide complementary and multilevel quantitative tools for assessing cortical E/I balance, network dynamics, and oscillatory responses. This review indicates that time-domain TEP components (e.g., N15, P30, N45) capture causal cortical responses to external perturbation with millisecond temporal resolution, extending beyond conventional EEG approaches that primarily record endogenous or sensory-evoked activity. GMFP and LMFP enhance robustness by integrating multichannel signals, thereby reducing the impact of polarity reversals at single electrodes and local noise on interpretation. In parallel, time-frequency measures (e.g., TRSP and ITPC) reveal transient oscillatory modulation induced by TMS, including power enhancement or suppression and changes in cross-trial phase synchronization, offering a spectral perspective on post-stimulation network evolution (Wang et al., 2024).

The complementarity of these metrics is most evident when they are interpreted within a shared temporal and spatial framework. TEPs provide a waveform-level description of phase-locked cortical responses, GMFP and LMFP summarize the global or local magnitude of these responses, and TRSP/ITPC reveal whether the same perturbation is expressed through oscillatory power modulation or phase alignment. Importantly, these measures should not be reduced to fixed one-to-one correspondences, such as assigning a specific TEP peak to a single frequency band. Rather, convergence across domains may strengthen physiological interpretation, whereas dissociation across domains may reveal whether a given effect is primarily expressed as local voltage amplitude, distributed network propagation, phase consistency, or non-phase-locked oscillatory activity. This cross-domain perspective is particularly important for clinical translation, where disease-related changes may appear differently across time-domain, spatial, and spectral measures (Casula et al., 2023; Ding et al., 2022; Hasanzadeh et al., 2019).

Despite these advances, key challenges continue to hinder the clinical translation of TMS-EEG. First, non-neural artifacts (e.g., scalp muscle activity and coil click-related auditory responses) substantially affect the reliability of early TEP components and high-frequency oscillatory measures. Although artifact-handling approaches such as ICA and SSP are widely used, differences across preprocessing pipelines can still yield 20–50% variability in GMFP/LMFP amplitude and may further distort TRSP and ITPC spectral patterns (Bertazzoli et al., 2021). Second, methodological heterogeneity, including stimulation intensity settings, coil orientation, and the choice of time-frequency decomposition methods, amplifies cross-study inconsistency. In particular, time-frequency outcomes may vary by approximately 20% as a function of algorithmic choice alone (Beck, Heyl et al., 2024; Bertazzoli et al., 2021). Third, inter-individual factors (e.g., age, cortical anatomy, and baseline brain state) further complicate interpretation. For instance, attenuated TRSP responses in older adults may reflect neurobiological changes associated with aging but may also be influenced by baseline oscillatory characteristics (Redondo-Camós et al., 2022; Morales-Torres et al., 2024). The literature broadly recognizes the promise of TMS-EEG for evaluating neuromodulatory effects; however, its utility as a diagnostic or predictive biomarker remains contingent on additional randomized controlled trials (RCTs), clearer cross-study comparability, and more consistent interpretation of variability arising from both methodological factors and individual differences (Cruciani et al., 2023).

Beyond these methodological and state-dependent factors, the interpretation of psychiatric TMS-EEG biomarkers should also acknowledge the structural architecture of the cortex. The induced electric field and the resulting evoked response are shaped not only by coil position and stimulation intensity, but also by local cortical folding, cortical thickness, tissue composition, and source-to-scalp geometry. Computational modeling studies have shown that gyral geometry and tissue properties can substantially affect the distribution of TMS-induced electric fields, indicating that the same nominal stimulation protocol may engage different cortical substrates across individuals (Thielscher et al., 2011; Opitz et al., 2011). This issue is particularly relevant in psychiatric and neurodevelopmental disorders, where structural MRI studies have identified common and disorder-specific cortical thickness alterations across internalizing, externalizing, and thought disorders, as well as morphometric signatures of internalizing–externalizing comorbidity (Yu et al., 2023; Kuang et al., 2023). Therefore, TMS-EEG abnormalities such as altered N100 amplitude, impaired inhibitory responses, or abnormal oscillatory modulation should not be viewed solely as functional markers detached from brain structure. Instead, they may reflect the interaction between local cortical morphology, developmental risk, synaptic physiology, and network-level dynamics. Incorporating individualized structural MRI, source modeling, and, where possible, electric-field estimation may help bridge electrophysiological readouts with the anatomical substrates that generate them.

From a mechanistic perspective, TEPs are generally considered to reflect the aggregate effects of excitatory and inhibitory postsynaptic potentials arising from populations of cortical pyramidal neurons and interneurons (Kirschstein and Köhling, 2009; Farzan et al., 2016), providing a conceptual basis for their use as proxies of E/I balance. This interpretation, however, requires caution when individual components are linked to specific neurotransmitter systems. Pharmacological TMS-EEG studies have provided important evidence that N45 is sensitive to GABA_A-ergic modulation and that N100 is influenced by GABA_B-ergic mechanisms, as reflected by the effects of benzodiazepine-type agents and baclofen on these components (Premoli et al., 2014; Premoli et al., 2017). Yet these associations do not necessarily indicate that each peak represents a direct readout of a single receptor system. For example, glutamatergic manipulation through NMDA receptor antagonism has also been shown to modulate N45 amplitude, suggesting that this component may reflect the dynamic balance between inhibitory and excitatory synaptic processes rather than GABA_A activity alone (Belardinelli et al., 2021). Similarly, although N100 is commonly discussed in relation to late inhibitory processing, its latency range overlaps with auditory and somatosensory evoked responses, and optimized sham-controlled work has shown that GABAergic modulation of N100 can also be observed under sensory-matched sham conditions (Gordon et al., 2023). These findings suggest that canonical TEP components are best interpreted as neurophysiologically informative but physiologically composite markers of cortical and network responses. This distinction is particularly relevant for clinical translation, because disease-related changes in TEP amplitudes may reflect overlapping contributions from receptor-level modulation, circuit-level E/I balance, sensory co-activation, baseline brain state, and methodological choices, rather than isolated abnormalities of a single neurotransmitter system.

At the network level, GMFP and LMFP provide complementary information by summarizing the spatial extent and magnitude of TMS-evoked responses. Their spatiotemporal evolution may reflect the propagation of stimulation effects through long-range connections and can be shaped by stimulation parameters such as pulse width and coil orientation (Casula et al., 2018). Stroke provides a useful example of why this propagation should be interpreted in relation to anatomical and network context. After unilateral stroke, TMS-evoked activity may depend on residual ipsilesional corticospinal pathways, transcallosal communication, and the recruitment of contralesional premotor or subcortical circuits. Stronger responses over the unaffected hemisphere should therefore not be viewed simply as maladaptive overactivity. Their functional meaning may vary with lesion severity and residual ipsilesional reserve: contralesional activity may interfere with ipsilesional reorganization in some patients, but may support recovery through compensatory pathways in others. This view is consistent with the bimodal balance-recovery framework, which proposes that the role of the contralesional hemisphere depends on lesion severity and residual structural reserve, and with recent evidence suggesting that contralesional premotor–subcortical interactions contribute to recovery in moderate-to-severe subcortical stroke (Di Pino et al., 2014; Fan et al., 2026). In this context, TEP propagation, GMFP/LMFP topography, and TRSP/ITPC dynamics may be more informative when interpreted together with hemispheric asymmetry and structural connectivity information, such as corpus callosum integrity or corticospinal tract reserve.

In parallel, TRSP and ITPC analyses further refine mechanistic interpretations of dynamic E/I regulation: high-frequency power enhancement is often associated with excitatory, glutamatergic-related processes, whereas low-frequency power suppression or synchronization changes are more commonly linked to GABAergic network resetting. Time-frequency analyses under LICI conditions suggest that suppressive effects spanning θ-β bands can extend to distant cortical regions, consistent with the presence of network-level E/I imbalance (Belardinelli et al., 2021; Takano et al., 2025; Mimura et al., 2025).

Importantly, much of the current evidence is derived from small-sample studies, which may inflate effect sizes and obscure within-disorder heterogeneity. A further translational barrier is the limited disease specificity of many TMS-EEG signatures. Reduced cortical inhibition, altered N45 or N100 responses, abnormal LICI-related suppression, and changes in oscillatory power are not confined to a single disorder, but have been reported across several neurological and psychiatric conditions, including schizophrenia, epilepsy, depression, and autism spectrum disorder (Farzan et al., 2010; Gefferie et al., 2023; Santoro et al., 2024; Takano et al., 2025; Mimura et al., 2025). This overlap does not diminish the mechanistic value of TMS-EEG, but it limits the use of isolated TEP components or single-band TRSP changes as stand-alone diagnostic biomarkers. In this sense, many TMS-EEG measures may currently be more appropriately interpreted as transdiagnostic markers of cortical reactivity, inhibitory regulation, or network propagation, rather than as disease-specific signatures.

Improving differential diagnostic specificity will likely require multivariate and multimodal approaches. Machine-learning models can integrate multiple TMS-EEG features, including TEP amplitudes and latencies, GMFP/LMFP topography, TRSP/ITPC patterns, trial-to-trial variability, and source-space estimates, rather than relying on a single component or frequency band. For example, machine-learning analysis of TMS-EEG features has shown promising classification performance in Alzheimer’s disease, suggesting that distributed time-domain features may provide more informative signatures than isolated peaks alone (Tăuƫan et al., 2023). However, such models require larger, independent, and preferably multicenter datasets to avoid overfitting and to establish generalizability. In parallel, multimodal integration with structural MRI, functional MRI, diffusion imaging, PET or fluid biomarkers may help determine whether a TMS-EEG abnormality reflects disease-specific pathology, compensatory network reorganization, medication effects, or a more general E/I imbalance. Therefore, future clinical TMS-EEG studies should move from single-marker interpretation toward cross-domain feature integration, external validation, and clinically meaningful differential classification.

Overall, the integrated use of TEPs, GMFP/LMFP, and TRSP/ITPC substantially enhances the potential value of TMS-EEG for mechanistic studies and clinical monitoring in neuropsychiatric disorders. However, an overreliance on static amplitude measures while neglecting dynamic features (e.g., waveform slope, cross-frequency coupling, or network coherence) may still limit the transition of TMS-EEG from a descriptive tool to a predictive modeling framework (Tremblay et al., 2019; Wang et al., 2024). Future work should aim for greater methodological consistency and clearer reporting, including transparent description of acquisition procedures such as RMT-based intensity calibration, coil positioning and orientation, trial number, and neuronavigation-guided targeting, while further integrating multimodal imaging (e.g., fMRI) and source reconstruction approaches to support translation from laboratory research to precision-medicine applications (Ilmoniemi and Kičić, 2010; Rogasch and Fitzgerald, 2013).

In summary, this review systematically synthesizes time-domain TEP analysis, spatially integrated GMFP/LMFP measures, and time-frequency TRSP/ITPC indices with respect to their roles in probing E/I balance, network dynamics, and biomarker development. These complementary metrics form a high-temporal-resolution, multilevel analytical framework that supports assessment in disorders such as epilepsy, Alzheimer’s disease, and depression (Darmani et al., 2019; Casula et al., 2023; Sheen et al., 2024). Although artifacts and methodological heterogeneity remain major barriers, ongoing improvements in reporting practices, cross-study comparability, and advanced preprocessing strategies may help alleviate these limitations and facilitate the evolution of TMS-EEG toward a more predictive clinical tool (Beck, Christiansen et al., 2024).

Looking forward, the combination of multimodal source-space analyses (e.g., sLORETA) and advanced artifact-management strategies (e.g., joint use of ICA and SSP) may further amplify the utility of TMS-EEG for evaluating neuromodulatory effects and E/I dynamics (Varoli et al., 2018; Moffa et al., 2022). Moreover, the development of real-time algorithms and AI-based approaches will provide a technical foundation for closed-loop TMS systems, enabling a shift from offline description toward real-time prediction. Finally, more comparable use and reporting of E/I-relevant time-frequency measures (e.g., frequency-band definitions and statistical strategies) may enhance comparability across disorders and centers and thereby support the clinical translation of TMS-EEG.
