# Correction: Toward clinical translation of TMS-EEG: an integrative review of multidimensional neurophysiological measures

**DOI:** 10.3389/fnhum.2026.1982339

**Published:** 2026-09-18

**Authors:** Wei Wang, Yuyao He, Haoyang Xing, Hongmei Zhang

**Affiliations:** 1 The Second Veterans Hospital of Sichuan, Chengdu, China; 2 Medical Imaging Laboratory, College of Physics, Sichuan University, Chengdu, China; 3 Huaxi MR Research Center (HMRRC), Department of Radiology, West China Hospital of Sichuan University, Chengdu, China

**Keywords:** clinical translation, global mean field power, time-frequency analysis, TMS-EEG, TMS-evoked potentials

Author Haoyang Xing was erroneously assigned to affiliation 1. The Second Veterans Hospital of Sichuan, Chengdu, China. This affiliation has now been removed for author Haoyang Xing.

Affiliation 2. Medical Imaging Laboratory, College of Physics, Sichuan University, Chengdu, China was omitted for author Haoyang Xing. This affiliation has now been added for author Haoyang Xing.

Author Hongmei Zhang was erroneously assigned to affiliation 2. Medical Imaging Laboratory, College of Physics, Sichuan University, Chengdu, China. This affiliation has now been removed for author Hongmei Zhang.

Adding:

Affiliation 1. The Second Veterans Hospital of Sichuan, Chengdu, China was omitted for author Hongmei Zhang. This affiliation has now been added for author Hongmei Zhang.

The original version of this article has been updated.

